# Exercise‐Induced cytokines, diet, and inflammation and their role in adipose tissue metabolism

**DOI:** 10.1002/hsr2.70034

**Published:** 2024-08-31

**Authors:** Abdullah Muataz Taha Al‐Ibraheem, Al‐Tuaama Abdullah Zeyad Hameed, Mohammed Dheyaa Marsool Marsool, Hritvik Jain, Priyadarshi Prajjwal, Istevan Khazmi, Ridha Saad Nazzal, Hussein Mezher Hameed AL‐Najati, Baqer Hadi Yusur Kharibet Al‐Zuhairi, Maryam Razzaq, Zainab Baqir Abd, Ali Dheyaa Marsool Marsool, Abdulrahman Isam wahedaldin, Omniat Amir

**Affiliations:** ^1^ Al‐Kindy College of Medicine University of Baghdad Jadirya Baghdad Iraq; ^2^ Privolzhsky Research Medical University, Nizhny Novgorod, Nizhny Novgorod Oblast Russia; ^3^ All India Institute of Medical Sciences Jodhpur India; ^4^ Bharati Vidyapeeth Medical College Pune India; ^5^ College of Medicine Hawler Medical University Erbil Iraq; ^6^ Al‐Kindy College of Medicine University of Baghdad; ^7^ College of Medicine University of Diyala, Baquba Iraq; ^8^ College of Medicine Kufa University Najaf Iraq; ^9^ College of Medicine Thi‐Qar University Nasiriyah City Iraq; ^10^ College of Medicine University of Baghdad Baghdad Iraq; ^11^ Al Manhal Academy Khartoum Sudan

**Keywords:** adipose tissue metabolism, diet, Exercise‐induced cytokines, inflammation, obesity

## Abstract

**Background:**

Obesity poses a significant global health challenge, necessitating effective prevention and treatment strategies. Exercise and diet are recognized as pivotal interventions in combating obesity. This study reviews the literature concerning the impact of exercise‐induced cytokines, dietary factors, and inflammation on adipose tissue metabolism, shedding light on potential pathways for therapeutic intervention.

**Methodology:**

A comprehensive review of relevant literature was conducted to elucidate the role of exercise‐induced cytokines, including interleukin‐6 (IL‐6), interleukin‐15 (IL‐15), brain‐derived neurotrophic factor (BDNF), irisin, myostatin, fibroblast growth factor 21 (FGF21), follistatin (FST), and angiopoietin‐like 4 (ANGPTL4), in adipose tissue metabolism. Various databases were systematically searched using predefined search terms to identify relevant studies. Articles selected for inclusion underwent thorough analysis to extract pertinent data on the mechanisms underlying the influence of these cytokines on adipose tissue metabolism.

**Results and Discussion:**

Exercise‐induced cytokines exert profound effects on adipose tissue metabolism, influencing energy expenditure (EE), thermogenesis, fat loss, and adipogenesis. For instance, IL‐6 activates AMP‐activated protein kinase (AMPK), promoting fatty acid oxidation and reducing lipogenesis. IL‐15 upregulates peroxisome proliferator‐activated receptor delta (PPARδ), stimulating fatty acid catabolism and suppressing lipogenesis. BDNF enhances AMPK‐dependent fat oxidation, while irisin induces the browning of white adipose tissue (WAT), augmenting thermogenesis. Moreover, myostatin, FGF21, FST, and ANGPTL4 each play distinct roles in modulating adipose tissue metabolism, impacting factors such as fatty acid oxidation, adipogenesis, and lipid uptake. The elucidation of these pathways offers valuable insights into the complex interplay between exercise, cytokines, and adipose tissue metabolism, thereby informing the development of targeted obesity management strategies.

**Conclusion:**

Understanding the mechanisms by which exercise‐induced cytokines regulate adipose tissue metabolism is critical for devising effective obesity prevention and treatment modalities. Harnessing the therapeutic potential of exercise‐induced cytokines, in conjunction with dietary interventions, holds promise for mitigating the global burden of obesity. Further research is warranted to delineate the precise mechanisms underlying the interactions between exercise, cytokines, and adipose tissue metabolism.

## 1 INTRODUCTION

The global prevalence of obesity is persistently increasing, irrespective of the implementation of evidence‐based public health standards. Committing to a healthy lifestyle is becoming progressively crucial in addressing this worldwide problem. Implementing calorie restriction, regular physical activity, or a combination of both has been widely acknowledged as a highly successful approach to preventing and managing obesity. Moreover, the advantages of consistent physical activity in reducing body weight are not just associated with a decrease in fat accumulation or the levels of lipids in the bloodstream. They are also connected to the production of proteins, peptides, enzymes, and metabolites that result from the contraction of skeletal muscles or other bodily organs.^1^, ^2^

The World Health Organization (WHO) recognizes that overweight is equal to or greater than 25 on the Body Mass Index scale (BMI) and obesity is to be equal to or more than 30 on the BMI Scale. In 2016, approximately 1.9 billion adults aged 18 and up were overweight, and about 650 million of these individuals were obese. Malnutrition is becoming a double burden in many low‐ and middle‐income nations.^3^

Understanding the interplay between adipose tissue accumulation, inflammatory cytokines, and their response to exercise in obese individuals is crucial for developing effective prevention strategies against obesity. Adipose tissue, considered an endocrine organ, plays a significant role in various diseases and responds to Interleukins (IL) and other metabolic signals.^4^, ^5^ Cytokines released by skeletal muscles during contraction are termed “myokines,” a concept introduced by Pedersen et al. in 2003.^6^ In contrast, cytokines originating from adipose tissue are referred to as adipokines. Additionally, mitokines are polypeptides released from mitochondria in response to stressors such as exercise. Myokines like irisin, IL‐6, IL‐15, METRNL, BAIBA, and myostatin, also hepatokines and specially FGF21, ANGPTL4, and follistatin, with adipokines like resistin, adiponectin, and leptin, are all involved in inflammatory processes for energy substrate redistribution and fat mass loss.^7^

## 2 METHODOLOGY

### 2.1 Literature search strategy

We conducted a methodical search for relevant literature by using various electronic databases such as PubMed, Scopus, Web of Science, and Google Scholar. The search strategy utilized a blend of keywords and controlled vocabulary terms pertaining to exercise‐induced cytokines, diet, inflammation, adipose tissue metabolism, and obesity. The search terms were appropriately combined using Boolean operators (AND, OR). The search was restricted to studies that were published in English, without any limitations on the date of publication.

### 2.2 Inclusion and exclusion criteria

Studies were considered eligible if they examined the impact of exercise‐induced cytokines on the metabolism of adipose tissue, either in animal models or human subjects. The inclusion criteria encompassed primary research articles, literature reviews, and meta‐analyses. The exclusion criteria included studies that only examined acute exercise responses, unrelated topics, or were not relevant to adipose tissue metabolism.

## 3 RESULTS AND DISCUSSION

### 3.1 Molecular mechanisms underlying Exercise‐Induced cytokine effect

Cytokines that are triggered by exercise are of significant importance in the regulation of adipose tissue metabolism, EE, thermogenesis, and the process of fat loss. The effects of these myokines, which are released from skeletal muscle as a result of physical activity, are mediated by many molecular processes and pathways. some examples of these mediators are:

#### 3.1.1 Interleukin‐6

IL‐6 is found to contribute to AMP‐activated protein kinase (AMPK) activation in humans. AMPK is an enzyme that plays a crucial role in systemic energy balance which increases several folds after exercise in both skeletal muscles and adipose tissue, the degree of the (AMPK) elevates with the intensity of the exercise, and this elevation plays a role in the adaptive response of the body and affected muscles, by altering muscles fuel reserves.^8^

#### 3.1.2 AMPK

AMPK is involved in a pathway that lowers the risk of obesity and insulin resistance by stimulating the oxidation of fatty acids. AMPK phosphorylates acetyl‐CoA carboxylase (ACC). The action of ACC is inhibited by this phosphorylation, which lowers the levels of malonyl‐CoA and this will disinhibit carnitine palmitoyltransferase 1 (CPT1) and increase oxidation of fatty acid, reducing lipid reserves in the liver and muscle.^9^, ^10^ AMPK also stimulates the uptake and release of lipids by phosphorylating lipases. In addition, it phosphorylates and inhibits transcription factors, most notably SREBP1 (sterol regulatory element binding protein 1), a key transcriptional lipid synthesis regulator.^11^

#### 3.1.3 Interleukin‐15

In course of vivo studies, it has been found that IL‐15 administration causes a 33% reduction in adipose tissue in experimental rats with no significant change in food intake behavior, the same result was observed in obese rodents in another vivo study of fat‐inhibiting IL‐15 which suggested the direct effect of IL‐15 in adipose tissue.^12^, ^13^

The observed pathway in these samples showed that IL‐15 upregulates PPARδ in brown adipose tissue (BAT) in rats. Subsequently, these receptors initiate the activation of Uncoupling Proteins (UCPs) and other proteins associated with the metabolism of fatty acids. This cascade of events leads to an increase in lipid catabolism and a decrease in lipogenesis.^14^ IL‐15 modifies lipid partitioning which results in less absorption into adipose tissue and more oxidation. When given to laboratory animals, IL‐15 partially inhibits the fatty acid synthesis in adipose tissue while promoting lipid oxidation. The suggested mechanism is PPARδ activated by IL‐15 acting as an agonist.^15^ PPARδ in turn has been known to be effective in increasing fatty acid oxidation in skeletal muscles and cultured myotubes.^16^

#### 3.1.4 Brain‐Derived neurotrophic factor

Brain‐Derived Neurotrophic Factor (BDNF) is produced in skeletal muscles, during exercise and muscle contractions. BDNF rises due to muscle electrical stimulation and this process was detected by increasing BDNF mRNA expression in the circulatory system. This elevation appears to increase fat oxidation by AMPK‐dependent pathway both in vivo and in vitro, exerting its effect within skeletal muscles, most likely via autocrine and/or paracrine pathways.^17^ In addition to the previous effects which were induced by regular exercise and muscle contraction, BDNF administration into the hypothalamic paraventricular nucleus has been shown to decrease food intake which results in decreasing body weight. This result comes through stimulation of the expression of uncoupling protein 1 (UCP‐1) in BAT, and increases the resting metabolic rate (RMR).^18^

#### 3.1.5 Irisin

The irisin hormone is created by proteolytic processing of a type I membrane protein that the Fibronectin type III domain‐containing protein 5 (FNDC5) gene encodes. Exercise, such as cycling, raises Peroxisome proliferator‐activated receptor‐gamma coactivator (PGC‐1) expression. The rise in PGC‐1 triggers the expression of FNDC5. Thus, exercise induces irisin, which causes the subcutaneous adipose tissue to undergo significant changes that stimulate the expression of UCP1 and browning. Fndc5 induces the expression of the transcription factor Peroxisome proliferator‐activated receptor alpha (PPAR‐α), which plays an important role in the production of UCP1 and other genes involved in the browning of adipose cells.^19^ Therefore, thermogenesis is stimulated by this expression increasing the number of brown adipocyte‐like cells and the expression of genes specific to those cells within the WAT.^19^, ^20^ Hence, irisin significantly raises total body EE and prevents the insulin resistance linked to obesity.^21^

#### 3.1.6 Myostatin

Myostatin which is produced by skeletal muscles in adult humans, seems to influence mitochondrial activity, AMPK activation, PPAR signaling, cyclooxygenase‐2 (COX‐2) production, and the transformation of WAT into a BAT‐like phenotype, which is an important thermogenesis regulator.^22^ BAT has a notable abundance of UCP 1, which serves the purpose of decoupling oxidative phosphorylation. This decoupling process leads to the liberation of surplus energy in the form of heat, hence facilitating the maintenance of the body's normal temperature. Hence, the absence of myostatin promotes heightened peripheral tissue fatty acid oxidation and increasing levels of Brown‐like adipose tissue in WAT. In myostatin‐deficient mice or when myostatin is antagonistic, these systems work together to promote EE and prevent obesity.^22^ Furthermore, it has been revealed that myostatin has the ability to suppress the process of adipogenic differentiation in both 3T3‐L1 and C3H 10T1/2 cells when cultured. This finding implies that myostatin may also directly impact adipocytes, as seen by the significant reduction in fat deposits reported in mice overexpressing myostatin.^23^, ^24^

#### 3.1.7 Fibroblast growth factor 21

The administration of FGF21 in murine subjects resulted in a notable decrease in the levels of leptin mRNA expression, whereas no significant impact was observed on the expression levels of adiponectin mRNA. The intervention demonstrated significant improvement in hepatic steatosis, accompanied by a notable reduction in epididymal fat. The present study investigated the underlying mechanism of fat loss, specifically focusing on lipolysis‐related molecules and blood free fatty acids (FFAs). It was shown that treatment with FGF21 resulted in an upregulation of the phosphorylation of Adipose Triglyceride Lipase and Hormone‐sensitive lipase at ser660.^25^

A further investigation showed that the introduction of FGF21 in mice resulted in a reduction in the levels of circulating glucose, insulin, cholesterol, triglycerides, FFAs, and leptin, which was dependent on the dosage administered. Additionally, FGF21 treatment decreased the expression of PPAR and its target genes adipocyte fatty acid binding protein‐2 (aP2) and CD36. Also, brown adipocytes’ levels of UCP‐1 and ‐2 mRNA expression increased, suggesting that BAT adaptive thermogenesis may have also had a role in the increased whole‐body EE following FGF21 treatment.^26^ Cold exposure induces an increase in sympathetic nerve activity (SNA) towards BAT, leading to the secretion of FGF21 and the initiation of thermogenesis. Concurrently, there is an increase in SNA towards WAT, resulting in the activation of the autocrine function of FGF21 in adipose tissue. Consequently, this mechanism triggers the synthesis of the cytokine CCL11, which serves to recruit eosinophils and macrophages, thereby facilitating the process of subcutaneous WAT beiging and consequently intensifying adipose tissue thermogenesis.^27^

#### 3.1.8 Follistatin

There is not much literature regarding FST effect on adipose tissue metabolism, but based on the known function of FST, which binds to the C‐terminal dimer of myostatin and prevents it from interacting to receptors,^28^ and its function in binding to, and inhibiting the activity of the superfamily of TGF‐β,^29^ FST may play a role in adipose tissue metabolism as both myostatin and TGF‐β play a role in adipose tissue metabolism and obesity.^22^, ^30^, ^31^, ^32^ In addition, brown preadipocyte development induces FST, and FST protein therapy during the differentiation of adipocyte‐raised mRNA and protein levels of thermogenic genes that are distinctive of brown and beige adipocytes. Furthermore, mice's BAT exposed to cold significantly increased the expression of FST.^29^

#### 3.1.9 Angiopoietin‐like 4

The activity of LPL is inhibited by ANGPTL4, thus playing a role in the regulation of the uptake of fatty acids produced from triglycerides by BAT during the course of a day. LPL is modulated by ANGPTLs at the protein level but not at the mRNA level. Several studies proposed the mechanism by which ANGPTL4 inhibits LPL, the first explanation for ANGPTL4's ability to inhibit LPL was that it could break down catalytically active LPL dimers into inactive LPL monomers.^33^ One study showed that the primary molecular mechanism for the suppression of LPL activity by ANGPTL3 and ANGPTL4 is identified as a 12‐amino acid consensus motif inside the coiled‐coil domain (CCD) of both proteins. This motif is found in the first‐helix predicted in the secondary structure and is unique to ANGPTL3 and ANGPTL4 and lacking in other ANGPTL family members.^34^ Other study showed that ANGPTL4 could bind to and inactivate LPL on endothelial cell surfaces when it is complexed with GPIHBP1.^35^ Recently, there has been a proposal suggesting that ANGPTL4 selectively inhibits LPL by binding to the lipid domain and then obstructing substrate catalysis at the active site.^36^ The overexpression of ANGPTL4 has been observed to decrease LPL activity, resulting in elevated levels of triglycerides in the bloodstream. Conversely, ANGPTL4 knockout mice have shown increased plasma LPL activity, enhanced clearance of triglycerides (TAGs), and reduced levels of TAGs in the plasma.^37^ Deletion of Angptl4 in the liver enhances metabolic function in response to high‐fat diet (HFD)‐induced obesity by boosting β‐oxidation through the activation of AMPK upon Angptl4 deletion.^37^

#### 3.1.10 Meteorin‐like (Metrnl)

High‐intensity exercise showed an increased mRNA expression in skeletal muscles.^38^ The increase in circulating Metrnl resulted in significant improvements in the overall thermogenic and mitochondrial gene programs of both subcutaneous and epididymal WAT. The genes UCP‐1, DIO2, PGC1‐α, and ERR‐α are among those that are included. In addition, there was a moderate upregulation of thermogenic gene programs observed in BAT. Moreover, the upregulated expression of Metrnl was found to induce increased mRNA levels of genes related to β‐oxidation, including Acsl1, Acox1, and CPT1. A reduction in body weight accompanied these improvements in thermogenic gene expression when compared to control mice. Additionally, the induction of Metrnl expression triggered the upregulation of IL4/IL13 expression, which played a pivotal role in initiating a cascade of events leading to increased expression of UCP‐1 and other thermogenic genes in adipose tissue. This increase in IL4/IL13 expression was a result of an augmentation in adipose tissue eosinophils. Consequently, this led to a transient increase in the browning of adipose tissue.^39^ Besides that, overexpression of Metrnl increases the expression of PPARγ which plays a huge role in adipocyte differentiation and insulin sensitization.^40^, ^41^, ^42^, ^34^, ^35^, ^36^, ^37^

#### 3.1.11 Β‐aminoisobutyric acid (BAIBA)

PGC‐1α stimulates the expression of the genes for the metabolic enzymes needed in myocytes for the synthesis and transport of BAIBA both in vitro and in vivo, BAIBA dramatically raises the expression of PPARα in white adipocytes. PPARα is known to promote the expression of UCP‐1, thus increasing thermogenesis and browning gene expression.^43^ BAIBA is associated with higher plasma leptin levels and fatty acid oxidation in mice.^44^ Activating PPARα by BAIBA increases hepatic fatty acid oxidation and decreases hepatic lipogenesis.^45^ In addition, the oxidation‐related genes of fatty acids including CPT1, acyl‐CoA oxidase, and fatty acid binding protein 3 (Fabp3) were considerably increased by BAIBA.^46^ Furthermore, BAIBA leads to phosphorylation of AMPK^47^ which, by phosphorylation and transcription factor deactivation, lowers the expression of fatty acid synthase (FAS) enzymes SREBP‐1c.^11^, ^16^

### 3.2 The impact of diet on adipose tissue

One of the initial approaches to address excessive body fat (BF) and prevent obesity is the adoption of a dietary regimen coupled with heightened physical activity. This is due to the fact that the principal contributors to obesity are rooted in a state of positive energy balance, wherein energy intake surpasses EE.^48^ While the general importance of diet composition and energy intake in relation to metabolism and energy balance has been highlighted,^49^ there remains a lack of consensus on the molecular adaptability of adipose tissue and the extent of weight loss resulting from different diets.^50^

The relationship between dietary carbohydrate (CHO) intake and the excessive production of insulin is widely recognized as a significant factor in the accumulation of BF. This is commonly referred to as the CHO‐insulin model of obesity.^51^ According to the model, it is predicted that a diet that is rich in CHO is likely to induce an elevation in insulin production. This increase in insulin levels is expected to inhibit the release of fatty acids into the bloodstream, thus leading to an augmentation in the accumulation of fat.^51^, ^52^ Furthermore, the reduced accessibility of fatty acids to metabolically active tissues leads to a condition of cellular hunger. Possibly due to an elevated ratio of cellular adenosine triphosphate to adenosine monophosphate there is an observed adaptive decrease in EE and an increase in food consumption.^53^, ^54^, ^55^ Therefore, it is hypothesized that the development of obesity is associated with a positive energy balance, which is attributed to insulin‐mediated mechanisms that promote fat accumulation and inhibit fat oxidation, mostly due to an elevated intake of dietary CHO.^56^ In this particular situation, it is likely that diets that effectively inhibit the increase in postprandial blood glucose levels (low glycemic index) confer metabolic advantages. Therefore, one potential effective strategy for preventing or reducing the danger of the CHO‐insulin model of obesity is to decrease the quantity of CHO in the diet.^57^, ^58^ A potential strategy to reduce insulin secretion, enhance adipose tissue fat mobilization, and facilitate the oxidation of FFAs involves reducing dietary CHO intake while increasing fat consumption. Consequently, these metabolic alterations may ultimately lead to a decrease in appetite, as well as an augmentation in BF loss and EE.^56^, ^59^

One further variant of the low‐CHO, high‐fat (LCHF) diet is known as the ketogenic diet (KD). This particular dietary approach involves a significant reduction in CHO consumption, while simultaneously maintaining a reasonable protein intake and ensuring that at least 70% of a person's daily caloric intake is derived from healthy fats.^57^ KD first emerged during the 1920s with the intention of replicating the physiological effects of fasting and providing therapeutic benefits for epilepsy.^60^, ^61^ Additionally, KD has been employed as a dietary intervention technique to promote weight loss and manage type 2 diabetes (T2D).^62^, ^63^ According to recent studies, KDs have been identified as a potentially safe and effective approach for enhancing metabolic regulation and facilitating weight loss.^64^, ^65^, ^66^ Moreover, it has been observed that KDs can also contribute to the enhancement of the conversion of FFAs into ketone bodies. This effect is achieved by a reduction in CHO metabolism and an increase in lipid oxidation.^62^, ^63^

Additionally, there have been reports indicating that KDs may potentially result in some adverse effects, including but not limited to headaches, lethargy, constipation, and muscle cramps, particularly during the initial stages of dietary adaptation.^57^, ^63^ Table 1 presents a comprehensive compilation of food items that are advocated for consumption within the context of a ‘Banting’ diet, which is a widely embraced LCHF dietary regimen.

**Table 1:** Recommended foods on a low‐carbohydrate high‐fat diet. LCHF dietary regimens advocate for the consumption of nutrient‐rich meals, including omelets, salads, and animal‐derived protein sources such as steak, fish, or chicken, accompanied with a variety of vegetables (57**).

Animal protein	Dairy	Fats	Nuts and seeds	Vegetables
Eggs Meats Poultry Game Seafood	Cottage cheese Cream Full‐cream Greek Yogurt Cheeses	Olive oil Avocados Coconut oil Macadamia nut oil	Almonds Flaxseeds Macadamia nuts Pecans Pine nuts	All green leafy vegetables, cruciferous vegetables or above ground vegetables

A prevalent worry voiced by medical professionals, is that any increase in dietary fat intake while following the LCHF diet will raise the chance of developing cardiovascular disease in the future.^67^ Original seven‐country study and the subsequent creation of the LFHC dietary recommendations to avoid cardiovascular disease are two major influences on this conviction. However, data from many meta‐analyses and randomized controlled trials (RCTs) indicates that LCHF diets consistently produce more positive improvements than LFHC diets in a number of cardiovascular risk factors. This is particularly relevant to those with diabetes, insulin resistance, AD, or NAFLD.^68^

CHO induces the biggest and longest‐lasting rises in blood glucose and insulin concentrations of any macronutrient.^69^ Therefore, it should come as no surprise that before the discovery of insulin, all diabetic patients, regardless of type, were advised to follow a CHO‐restricted diet that was frequently accompanied by fasting or even starvation. Again, being suggested as a viable first‐line therapy for T2DM nowadays are LCHF diets.^57^

A non‐calorie‐restricted low‐fat low‐calorie diet (LLD) in individuals with T2D significantly improves glycemic control and body composition without adversely affecting cardiovascular risk factors. In relation to conventional cardiovascular risk factors and hypoglycemia, it seems that implementing a CHO restriction to 10–25% E% of one's diet is a viable and safe nutritional approach.^64^, ^65^

### 3.3 Nutritional modulation of adipose tissue metabolism

The mechanism of adipogenesis is pivotal in the development and regulation of adipose tissue. There are multiple factors that can affect such mechanism:

#### 3.3.1 Dietary compounds that promote adipogenesis & lipid storage

*CHO* can impact the formation of adipose tissue, promoting adipogenesis. They have been implicated in obesity through some mechanisms**.** First, glucose itself serves as a critical substrate for adipogenesis, this glucose can be taken up by preadipocytes and used as an energy source for the process of differentiating into mature adipocytes,^70^ Second, insulin signalling, one of its primary roles is to facilitate the uptake of glucose by cells, including preadipocytes, it should be noted that the drug metformin can also induce this process by increasing insulin sensitivity, especially when taken with insulin,^71^ Insulin also promotes the conversion of glucose into stored fat (lipogenesis) by regulating transcriptional factors like [SREBP‐1].^72^ Complex CHO, such as whole grains, most vegetables and kidney beans provide a slower and more sustained release of glucose into the bloodstream (low glycemic index or GI), which may have a less pronounced effect on adipose tissue formation compared to simple CHO like sugar, which can lead to rapid spikes in blood sugar levels and insulin release.^71^

Saturated fatty acids (SFAs), promote adipogenesis and lipid storage by activating pathways that increase the differentiation of precursor cells into mature adipocytes, facilitating the storage of excess fats within these cells, their effects on this process are accomplished by certain mechanisms: *A)* SFAs can trigger low‐level inflammation in the body. Chronic inflammation can disrupt normal metabolic processes and promote the accumulation of fat in adipose tissue. There is substantial evidence indicating that consuming high‐fat meals can facilitate the movement of endotoxins (such as lipopolysaccharide or LPS) into the blood.^73^ This, in turn, activates innate immune cells, resulting in a temporary post‐meal inflammatory response.^73^ SFAs also activate toll‐like receptors. This activation is believed to stimulate the production of pro‐inflammatory cytokines like TNF‐α through the COX‐2 and NF‐κβ pathways,^74^ furthermore SFAs can induce some inflammatory markers like [Kyn]: [Trp] ratio and C‐reactive protein (CRP).^75^ *B)* A diet high in SFAs has been associated with insulin resistance, a study^76^ supplies substantial proof that FFAs diminish insulin sensitivity leading to insulin resistance, which stimulates the conversion of excess glucose into fat and promotes adipogenesis. *C)* Saturated fats mainly influence the secretion of hormones involved in fat regulation, like Adiponectin (also called Acrp30), Adiponectin circulates at relatively high (mg/L) concentrations,^77^ the functions of adiponectin promotes insulin sensitivity (Adiponectin exerts its insulin‐sensitizing influence by binding to its receptors AdipoR1 and AdipoR2),^78^, ^79^ regulation of fat metabolism and anti‐inflammatory properties, feeding rodents a HFD results in reduced secretion of Acrp30,^78^, ^80^ Thus, low concentrations of adiponectin in the setting of HFD lead to insulin resistance and altered adipose tissue metabolism that promote adipogenesis.

#### 3.3.2 Dietary compounds that inhibit adipogenesis and lipid storage

There are multiple dietary compounds that have been investigated for their direct anti‐adipogenic properties. These compounds are good diets to reduce weight; they include (certain facts, polyphenols and capsaicin), and some have an indirect reduction in adipogenesis (fibers and proteins). Polyphenols are natural compounds found in a variety of plant‐based foods, such as fruits, vegetables, tea, and red wine. Some polyphenols have been studied for their potential to inhibit adipogenesis, such as green tea extract which has been shown to inhibit adipogenesis by reducing the expression of adipogenic transcription factors like PPAR‐γ and C/EBPα.^81^

Certain fatty acids have been studied for their potential to inhibit adipogenesis, they include:

A) *Omega‐3 fatty acids (ω‐3PUFA)* are a type of polyunsaturated fatty acids (PUFAs), that have been shown to inhibit the differentiation of preadipocytes (precursor cells) into mature adipocytes,^82^ this type of PUFA induces fatty acid oxidation, thereby reducing fat accumulation,^83^, ^84^ *ω‐3PUFA* is known for their anti‐inflammatory properties,^85^ by reducing inflammation, omega‐3s may affect adipogenesis, as inflammation is linked to obesity and metabolic disorders, omega 3 also reduces insulin resistance^86^ a known contributor for adipogenesis.
B) *Conjugated Linoleic Acid (CLA) is* a type of fatty acid found naturally in the meat and dairy products of ruminant animals, CLA inhibits adipogenesis^87^ by reducing the expression of key adipogenic transcription factors such as PPAR‐γ and C/EBPα. It may also enhance fat oxidation and reduce fat storage.

*Monounsaturated fats*, found in foods like olive oil^88^ and nuts,^89^ have a lipolytic activity that is induced by adrenaline when examined in animals^90^

A) Capsaicin, the compound responsible for the spicy heat in chilli peppers, some studies suggest that capsaicin may inhibit the differentiation of 3T3‐L1 preadipocytes into mature adipocytes.^91^ furthermore, his anti‐inflammatory effect can reduce fat accumulation leading to a further decrease in adipogenesis,
B) in addition, dietary proteins and fibers can indirectly reduce adipogenesis by influencing various mechanisms related to appetite control, insulin sensitivity, and weight management, potentially reducing adipogenesis.
C) Proteins are highly satiating, protein‐rich foods can the induce release of hormones such as GLP‐1, leptin, and Peptide YY (PYY)^92^ which help you feel full and satisfied after meals, this can lead to reduced calorie intake and, over time, contribute to weight management. A lower calorie intake can limit the excess calories available for fat storage.

Fibers, a soluble fiber in fiber‐rich foods, can improve insulin sensitivity^90^ and slow CHO digestion^91^ and absorption, stabilizing blood sugar, and preventing rapid glucose spikes that can promote overeating and fat storage.

### 3.4 Thermogenesis and its effect on adipose tissues

The body's heat production through calorie burning for energy, is influenced by factors like activity, diet, and environment. In metabolism, it affects EE and weight regulation. An example of diet‐induced thermogenesis is a meal rich in protein, as protein has a high thermic effect, protein‐rich meals require extra energy for digestion.^93^ So, in general a high protein diet can increase the thermic effect of food (TEF) and satiety compared to carbohydrate‐rich meals.^94^

The body needs energy to digest, absorb, and metabolize the amino acids. This process activates the sympathetic nervous system (SNS) through the β‐adrenergic receptor (βAR), increasing EE, Norepinephrine released from sympathetic nerve terminals initiates BAT thermogenesis.^95^ It is found that mice with lower levels of βAR experienced a decrease in metabolic rate and showed a tendency towards obesity.^96^ Amino acids from proteins (tyrosine, e.g.) can also be converted into neurotransmitters, like norepinephrine, further stimulating the SNS.^97^ There are several thermogenic foods (stimulate BAT thermogenesis or stimulate the SNS, leading to BAT thermogenesis) apart from proteins, such as, caffeine‐containing foods, chilli peppers (capsaicin), ginger, and coconut oil, which contain medium‐chain triglycerides (TGAs).^98^, ^99^, ^100^

### 3.5 The effects of antioxidants and bioactive compounds, on adipose tissue function

The administration of ginger extraction in mice resulted in prolonged activation of the PPAR‐α pathway, which was associated with a reduction in diet‐induced obesity and an enhancement in exercise endurance capacity through the promotion of fat breakdown in skeletal muscle in vitro.^101^ The compound known as curcumin has been observed to effectively inhibit the activity of fatty acid synthase (FAS).^102^ This inhibition of FAS has been linked to curcumin's ability to limit the process of adipocyte development and the buildup of lipids. Therefore, curcumin is seen as having potential use in the prevention of obesity.^102^ The ingestion of black garlic extract has been observed to result in a decrease in the expression of SREBP‐1C mRNA. This reduction in expression subsequently leads to the downregulation of lipid and cholesterol metabolism. Consequently, the concentrations of total lipids, triglycerides, and cholesterol in the blood exhibited a decrease.^102^, ^103^

The compound known as alliin, derived from garlic, exerts control over the inflammatory state of adipocytes through various mechanisms.^102^ These include reducing the expressions of IL‐6 and MCP‐1 at both the mRNA and protein levels, as well as inhibiting ERK1/2 phosphorylation in LPS‐stimulated 3T3‐L1 adipocytes. Additionally, alliin induces an anti‐inflammatory gene expression profile in adipocytes and alters their metabolic profile.^104^ The administration of soy isoflavones demonstrated a notable suppression of metabolic alterations linked to obesity and inflammation. Additionally, it exhibited an enhancement in the oxidative stress and inflammatory responses of adipose tissue. These findings suggest that soy isoflavones, as a natural phytoestrogen, possess potential as an antioxidant and anti‐inflammatory agent for mitigating the metabolic consequences experienced by postmenopausal women.^105^

Resveratrol, a naturally derived diphenolic compound, has been observed to inhibit the conversion of glucose to lipids in adipocytes by attenuating the insulin effect. Furthermore, resveratrol enhances epinephrine‐triggered lipolysis, even in the presence of insulin, through a synergistic interplay between resveratrol and epinephrine.^106^ The supplementation of purified sweet cherry anthocyanins (CACN) has been found to potentially decrease adipocyte size, leptin secretion, serum glucose levels, triglyceride levels, total cholesterol levels, LDL‐cholesterol levels, and liver triglyceride levels.^107^ In addition, it has been observed that the administration of CACN leads to a significant decrease in the expression levels of IL‐6 and TNF‐α genes, while simultaneously causing a notable rise in the activity of SOD and GPx. The administration of grape‐seed procyanidins (GSPE) has been found to decrease the expression of IL‐6 and monocyte chemoattractant protein‐1 (MCP‐1), while simultaneously increasing the synthesis of the anti‐inflammatory adipokine adiponectin^108^ These findings imply that GSPE may potentially provide positive effects on low‐grade inflammatory conditions such as obesity and T2D. The ingestion of wild blueberries led to a reduction in plasma levels of TNF‐α, IL‐6, and CRP while increasing the concentration of adiponectin. In addition, the expression levels of IL‐6, TNF‐α, and NF‐kB were observed to be decreased in both the liver and the abdominal adipose tissue. However, the expression of CRP was found to be downregulated solely in the liver.^109^

### 3.6 The effect of exercise on fatty acid composition in adipose tissue

The condition of having excessive body weight is caused by an imbalance between the amount of energy consumed and the amount of energy expended. One of the strategies employed to uphold a healthy body weight and promote lipid catabolism is the augmentation of physical activity. with different approaches to later such as simple physical activities as walking rather than using motorized alternatives (ie, automobiles, elevators, and escalators) whether it is continuous or intermittent with personal variation regarding the time of where results were clear to the participants.^110^, ^111^

**Figure 1:**
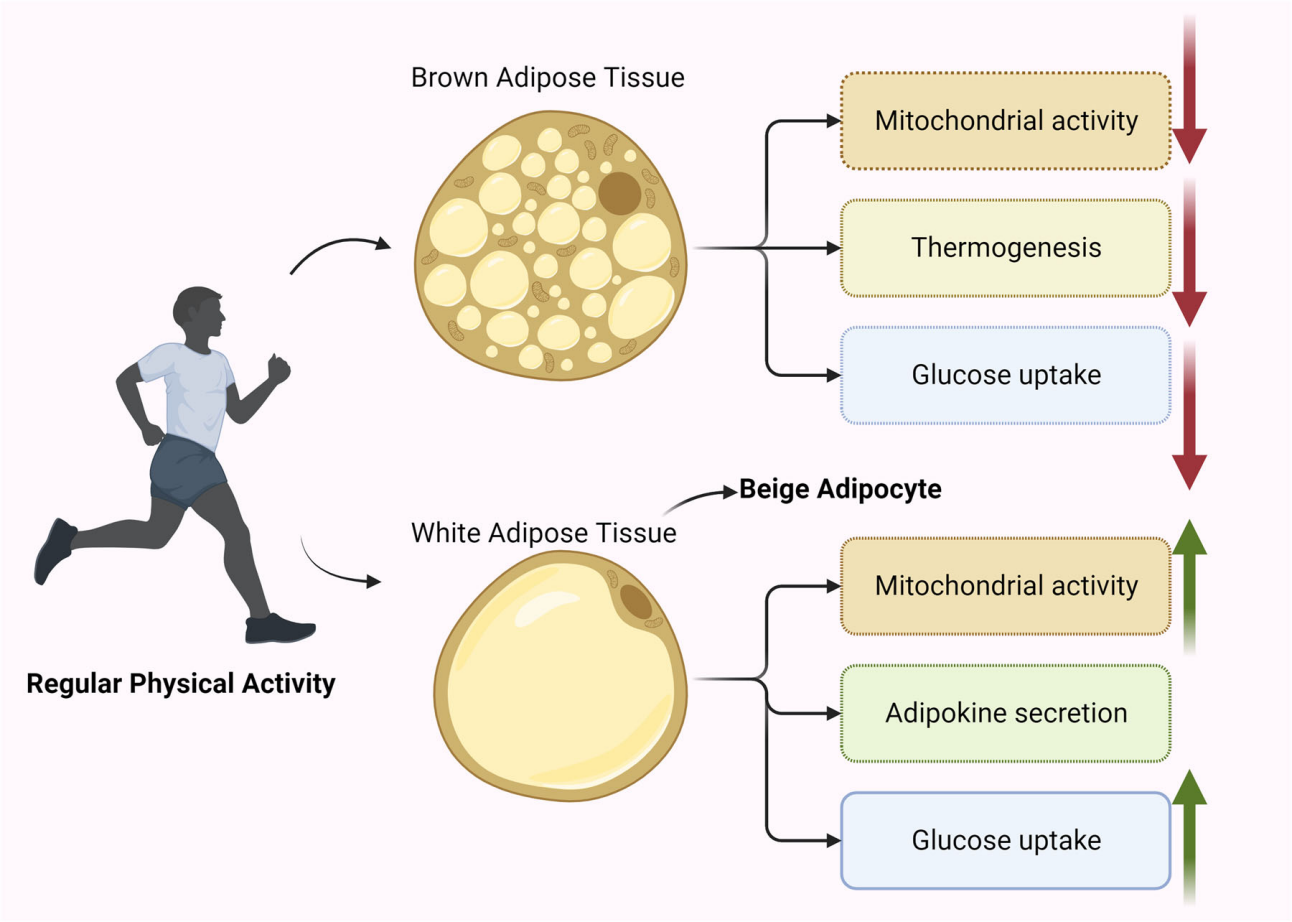
illustrates the physiological changes that occur in white adipose tissue (WAT), brown adipose tissue (BAT), and beige adipocytes in response to exercise.

This process entails the decomposition of triacylglycerols present in AT, the subsequent liberation of FFAs into the bloodstream, and the facilitation of their oxidation in various tissues, including skeletal muscles. In addition, physical activity plays a role in augmenting the quantity of mitochondria in WAT and promoting the activation of genes related to brown adipocytes. This process, known as “beiging” of WAT, has been observed to improve glucose intolerance caused by a HFD (Figure 1).^43^, ^112^, ^113^

The impacts of physical exercise on AT are correlated with notable alterations in metabolism and FA composition. The emergence of beige AT is a result of the process known as “beiging” of white adipocytes, wherein these cells undergo a phenotypic and metabolic transformation to resemble brown adipocytes. WAT plays a crucial role in the storage of triacylglycerols and the release of different adipokines into the bloodstream.^114^ In rodents, BAT is predominantly found in a sizable interscapular depot, along with smaller depots in other locations. the presence of BAT in humans, primarily in regions surrounding the neck, spine, and major blood arteries. The BAT, which possesses a high concentration of mitochondria, plays a vital role in the process of thermogenesis.^115^ Controversial findings have emerged from recent studies on both animals and humans conducted on both humans and animals on the impact of regular physical exercise on BAT activity. A reduction in BAT activity, including mitochondrial activity, glucose absorption, and thermogenesis, has been documented in individuals who have undergone training.

The composition of AT lipids relays on exercise intensity.^111^ Most AT lipids are triacylglycerols, making them the most abundant class of lipids.^111^ Studies showed that chronic exercise increases linoleic acid and decreases oleic acid in the subcutaneous WAT.^112^, ^113^ Since metabolic disorders are associated with increased oleic acid synthesis, the postexercise decrease in AT oleic acid content (which was attributed to decreased stearoyl‐CoA desaturase activity in AT) is considered a positive alteration.^113^ In addition, new research suggests that physical activity may improve the preferential mobilization of fatty acids from AT. When compared to untrained control, the authors found a significant decrease in triacylglycerols and cholesterols, as well as alterations in the fatty acid profile of AT, including a decrease in palmitoleic acid and an increase in stearic acid.^114^ An investigation observed a significant increase in linoleic acid in obese elderly subjects following 6 months of increased physical activity, while there was no such effect in untrained controls.^116^ Collectively, these scant human studies indicate that chronic exercise may decrease oleic acid content while simultaneously increasing linoleic acid. Due to the prevalence of oleic acid in triacylglycerols, a decrease in its content contributes to an increase in other fatty acids.

A study examined the effect of a 3‐week exercise training program on the fatty acid composition of phospholipids and triacylglycerol from subcutaneous WAT and BAT in mice).^117^ The study revealed a decrease in PUFA and an increase in monounsaturated fatty acids (MUFA) in relation to WAT. On the contrary, there was a decrease in MUFA and an increase in PUFA in BAT. Consequently, BAT responds differently to exercise than WAT in terms of phospholipid fatty acid composition.^118^, ^119^ The study also revealed that exercise decreased the content of triacylglycerol in WAT and BAT (i.e., the amount of fat stored in these tissues decreased), whereas there was an increase in the level of triacylglycerol containing long chain fatty acids in both BAT and WAT, indicating that exercise caused a shift of triacylglycerol toward longer chain fatty acids.^118^

### 3.7 Diet strategies to prevent obesity and improve adipose tissue health

#### 3.7.1 Restriction of a specific food group: Plant‐Based diets

Although plant‐based diets (PBDs) are low in total fat, saturated fat, and cholesterol, they are rich in unsaturated fatty acids and fibers.^120^ Therefore, they have been shown to have a significant role in enhancing blood lipid profile and adiposity.^121^ The Mediterranean diet, vegan and vegetarian diets,^122^ and the Nordic diet^123^ are among the many plant‐based dietary patterns. These dietary patterns also include eggs, dairy products, and fish, but almost no meat. Studies demonstrated the efficiency of PBDs for weight loss.^122^ Additionally, when compared with other energy‐restricted regiments, PBDs provide greater nutritional value.^121^ Studies indicate that PBDs have a positive impact on BMI and contribute to the reduction of chronic diseases.^121^, ^124^ Based on the Adventist Health Study‐2, those who refrain from consuming animal products have the lowest mean BMI, whereas meat eaters have the highest mean BMI.^125^ Likewise, a recent study revealed a significant decrease in BMI after six months of committing to a PBD routine without caloric restrictions, as opposed to a minimal change in BMI among obese individuals receiving standard care.^126^ The process linking these benefits to PBDs involves alteration in satiety, and inflammation.^127^ Further analysis revealed that PBDs are associated with a reduction in the inflammatory profiles typically associated with obesity, implying that they could be used for the therapeutic prevention of chronic diseases.^127^ Some types of PBDs also contain anti‐inflammatory bioactive compounds (chemical substances that have a direct and measurable effect on the living organism).^128^ fruits, nuts, and seeds are abundant in bioactive substances. Recent in vivo and in vitro studies have shown that food containing bioactive compounds can influence gene expression in AT, proposing that these foods may be useful for preventing obesity and metabolic disorders.^129^, ^130^

#### 3.7.2 Intermittent fasting and its effect on adipose tissue

In the past few years, periodic and recurring energy restriction strategies, such as intermittent fasting (IF), have acquired popularity as an alternative method of weight loss. IF involves going without food and drink with caloric content for set periods of time, followed by periods of normal eating. Fasting durations and how often they occur can vary among IF approaches. Exercise programs and other dietary approaches are compatible with IF. Periodic fasting (often known as the 5:2 diet), alternate‐day fasting, and religious fasting are popular forms of IF. Fasting can alter cellular and metabolic pathways.^131^ The most notably documented physiological advantages of IF are decreased blood pressure, body fat, fasting glucose, inflammation, and improved insulin sensitivity^131^ (131,. Research has shown that IF is equally efficacious as continuous calorie restriction in reducing body weight and risk factors associated with metabolic diseases among young, overweight women.^132^ According to those studies, IF decreased body weight by 3–8% and 4–14%, respectively, after 3–24 weeks and 6–24 weeks.^133^ The effects of intermittent and continuous caloric restriction on adipose tissue and gene expression were investigated over the course of fifty weeks. Intermittent calorie restriction may be comparable to continuous calorie restriction for weight loss, but not superior.^134^ It's also important to note that the short‐term negative effects of fasting, like tiredness, headaches, and constipation, might be similar to keto diet and depend on the duration of the fasting period.^132^ Also, studies demonstrate that IF improves subjects’ eating habits and mood who are overweight or obese.^132^, ^135^

## 4 EXERCISE MIMETICS

Exercise mimetics are a proposed class of therapeutics that specifically mimic or enhance the therapeutic effects of exercise.^136^

### 4.1 AMPK activators

Among the most promising compounds that mimic exercise are those that activate AMP‐activated protein kinase (AMPK), which reacts immediately to exercise‐induced energy stress. It is well documented that 5‐aminoimidazole‐4‐carboxamide ribonucleotide (AICAR) induces glucose uptake in muscles and fatty acid oxidation when administered to rats.^137^ AICAR treatment of sedentary rats induces a molecular cascade that promotes mitochondrial biogenesis and fatty acid oxidation, which is analogous to the effects of exercise.^138^ Therefore, this intervention improves exercise endurance, insulin sensitivity, and prevents diet‐induced adiposity in these mice.^139^ Intracellular conversion of AICAR to ZMP (an endogenous metabolite that allosterically activates AMPK) induces AMPK activation. Recently synthesized small molecule Compound 14 increases intracellular ZMP and activates AMPK. Analogous to AICAR, the administration of Compound 14 to obese rodents results in reduced weight gain and enhanced glucose tolerance, thereby validating the advantageous effects of AMPK activation on health.^140^

### 4.2 SIRT1 activators

Resveratrol, a natural substance extracted from red grape skins.^141^ Although resveratrol was first reported as a direct Sirtuin 1 activator, subsequent research demonstrated that it really activates Sirtuin 1 indirectly by increasing levels of its substrate NAD+ via the AMPK pathway.^142^, ^143^ Resveratrol administration to mice, induces Sirtulin1 in skeletal muscle, which in turn increases peroxisome proliferator‐activated receptor gamma coactivator 1 activity and ultimately activates Estrogen‐Related receptor α (EERα), ERRγ, and Peroxisome proliferator‐activated receptor δ. These genetic modifications collectively result in the upregulation of genes involved in mitochondrial biogenesis, fatty acid transport, and oxidative metabolism. Despite its modest potency, resveratrol has benefits, such as preventing diet‐induced obesity.^144^

### 4.3 REV‐ERBα ligands

In vivo purified proteins from HEK293T cells evaluations demonstrated that SR9009 and SR9011 are effective exercise simulators. They are synthetic ligands for the nuclear receptor REV‐ERBα, which regulates the circadian rhythm and energy metabolism. When present, REV‐ERBα inhibits the expression of its target genes. As the majority of muscle metabolic genes are time‐dependent, REV‐ERBα controls the relative amplitudes of these circuits.^145^ Exercise further stimulates the expression of REV‐ERBα in muscle, where it is specifically found in oxidative myofibers. This Overexpression increases mitochondrial and fatty acid metabolism, whereas its deletion decreases these processes. The method by which REV‐ERBα indirectly induces the expression of mitochondrial genes via the AMPK‐SIRT1‐PGC1a pathway is unknown, but it has been demonstrated that REV‐ERB does not regulate mitochondrial genes directly. Therefore, REV‐ERBα is a promising option for the development of exercise simulators.^142^

## 5 ESTROGEN‐RELATED RECEPTOR GAMMA LIGANDS (EERGAMMA)

Direct control of mitochondrial oxidative genes by Estrogen‐related receptor (ERR) makes it a potentially useful target for exercise mimetics. Significant upregulation of genes associated with mitochondrial biogenesis, fatty acid oxidation, and the tricarboxylic acid cycle (TCA cycle) is observed in mice primary muscle cells treated with the synthetic ERR agonist GSK4716. This finding indicates the presence of a direct effect of the agonist.^146^, ^147^

## 6 CONCLUSION AND FUTURE DIRECTIONS

Exercise‐induced cytokines, diet, and inflammation play a complex role in regulating adipose tissue metabolism. These factors can influence energy expenditure (EE), thermogenesis, fat loss, and adipogenesis. Understanding the mechanisms by which these factors interact is essential for developing effective obesity prevention and treatment strategies. Further research is needed to fully understand the complex interplay between exercise‐induced cytokines, diet, inflammation, and adipose tissue metabolism. This research should focus on identifying the specific mechanisms by which these factors interact, as well as developing more effective interventions to target these mechanisms. The findings of this study have several implications for obesity prevention and treatment. First, they suggest that exercise and diet can be effective strategies for reducing adipose tissue accumulation and improving metabolic health. Second, they suggest that targeting exercise‐induced cytokines, such as IL‐6 and irisin, may be a promising therapeutic approach for obesity. Third, they suggest that managing inflammation may be important for preventing and treating obesity.

## AUTHOR CONTRIBUTIONS

**Abdullah Muataz Taha Al‐Ibraheem**: Conceptualization; Resources; Visualization; Validation; Writing—original draft; Writing—review and editing. **Al‐Tuaama Abdullah Zeyad Hameed**: Resources; Validation; Conceptualization; Writing—original draft. **Mohammed Dheyaa Marsool Marsool**: Conceptualization; Resources; Visualization; Writing—original draft; Writing—review and editing. **Hritvik Jain**: Resources; Validation; Writing—original draft; Writing—review and editing. **Priyadarshi Prajjwal**: Validation; Writing—original draft; Writing—review and editing. **Istevan Khazmi**: Validation; Writing—original draft; Writing—review and editing. **Ridha Saad Nazzal**: Writing—original draft; Validation. **Baqer Hadi Yusur Kharibet Al‐Zuhairi**: Validation; Writing—original draft. **Maryam Razzaq**: Writing—original draft; Validation. **Zainab Baqir Abd**: Validation; Writing—original draft. **Ali Dheyaa Marsool Marsool**: Validation; Writing—original draft; Writing—review and editing. **Abdulrahman Isam wahedaldin**: Writing—original draft; Writing—review and editing; Conceptualization. **Omniat Amir**: Validation; Writing—review and editing.

## CONFLICT OF INTEREST STATEMENT

The authors declare no conflicts of interest, financial or otherwise.

## ETHICS STATEMENT

Not applicable.

## TRANSPARENCY STATEMENT

The lead author Omniat Amir affirms that this manuscript is an honest, accurate, and transparent account of the study being reported; that no important aspects of the study have been omitted; and that any discrepancies from the study as planned (and, if relevant, registered) have been explained.

## Data Availability

Data sharing is not applicable to this article as no new data were created or analyzed in this study.
